# Ultraviolet Absorption Cross-Sections of Ammonia at Elevated Temperatures for Nonintrusive Quantitative Detection in Combustion Environments

**DOI:** 10.1177/0003702821990445

**Published:** 2021-02-02

**Authors:** Wubin Weng, Shen Li, Marcus Aldén, Zhongshan Li

**Affiliations:** Division of Combustion Physics, 5193 Lund University, Lund, Sweden

**Keywords:** Ammonia, ultraviolet absorption spectroscopy, UV absorption spectroscopy, ultraviolet absorption cross-section, UV absorption cross-section, quantitative optical measurement, combustion, high temperature

## Abstract

Ammonia (NH_3_) is regarded as an important nitrogen oxides (NOx) precursor and also as an effective reductant for NOx removal in energy utilization through combustion, and it has recently become an attractive non-carbon alternative fuel. To have a better understanding of thermochemical properties of NH_3_, accurate in situ detection of NH_3_ in high temperature environments is desirable. Ultraviolet (UV) absorption spectroscopy is a feasible technique. To achieve quantitative measurements, spectrally resolved UV absorption cross-sections of NH_3_ in hot gas environments at different temperatures from 295 K to 590 K were experimentally measured for the first time. Based on the experimental results, vibrational constants of NH_3_ were determined and used for the calculation of the absorption cross-section of NH_3_ at high temperatures above 590 K using the PGOPHER software. The investigated UV spectra covered the range of wavelengths from 190 nm to 230 nm, where spectral structures of the $\overset{∼}{A}{\;}^{1}A{\prime \prime }_{2}\overset{∼}{X}{\;}^{1}A{'}_{1}$ transition of NH_3_ in the umbrella bending mode, *v*_2_, were recognized. The absorption cross-section was found to decrease at higher temperatures. For example, the absorption cross-section peak of the (6, 0) vibrational band of NH_3_ decreases from ∼2 × 10^−17^ to ∼0.5 × 10^−17^ cm^2^/molecule with the increase of temperature from 295 K to 1570 K. Using the obtained absorption cross-section, in situ nonintrusive quantification of NH_3_ in different hot gas environments was achieved with a detection limit varying from below 10 parts per million (ppm) to around 200 ppm as temperature increased from 295 K to 1570 K. The quantitative measurement was applied to an experimental investigation of NH_3_ combustion process. The concentrations of NH_3_ and nitric oxide (NO) in the post flame zone of NH_3_–methane (CH_4_)–air premixed flames at different equivalence ratios were measured.

## Graphical abstract

**Graphical Abstract:**
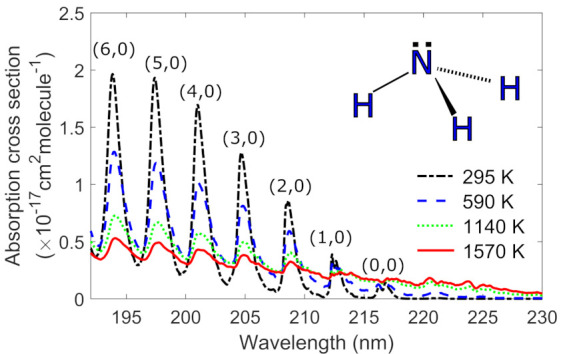

## Introduction

Ammonia (NH_3_) plays a significant role in energy field and attracts numerous studies of its thermochemical properties. Firstly, NH_3_ attracts increasing interests being regarded as a potential carbon-free alternative fuel.^1−4^ Thus, in the past few years, the combustion characteristics of NH_3_ were intensively investigated.^2,5−12^ Secondly, in the combustion of solid fuels, such as coal, biomass, and municipal solid waste, NH_3_ is an important precursor of nitrogen oxides (NOx),^13,14^ which is mostly released during their de-volatilization stage.^15^ Moreover, in solid fuel gasification, NH_3_ was regarded as an unwanted component of produced gas.^16^ Therefore, studies of the fate of NH_3_ during solid fuel thermal conversion processes are important. Thirdly, NH_3_ is widely used as a typical reductant in DeNOx techniques, such as selective non-catalytic reduction and selective catalytic reduction.^17^ To have a deep understanding of the chemical reactions involved in the aforementioned thermochemical processes, experimental studies with accurate in situ detection of NH_3_ are crucial. The concentration of NH_3_ under analysis could vary from below 100 to 10 000 parts per million (ppm), produced from solid fuel gasification at temperature around 1200 K^18^ or the combustion of NH_3_ at a temperature close to 2000 K.^2^ Numerous measurements have been conducted using Fourier transform infrared spectroscopy and chemical absorption techniques. However, these sampling-based techniques introduce unknown measurement uncertainties due to the high hygroscopicity and reactivity of NH_3_, and the intrusive processes hinder the possibility of reliable in situ measurements, especially in combustion environments. To have nonintrusive measurements of NH_3_ in hot gas environments, several optical diagnostics have been developed, such as broadband UV absorption spectroscopy,^19−21^ laser-induced photofragmentation fluorescence (LIPF),^22^ femtosecond laser-induced plasma spectroscopy,^23^ two-photon laser-induced fluorescence,^24−27^ degenerate four-wave mixing,^28,29^ and infrared absorption spectroscopy.^30−32^ Compared with the other techniques, UV absorption spectroscopy has some advantages. Firstly, it can achieve quantitative detection without calibration. Secondly, it has better species specificity than photofragmentation techniques. Thirdly, it has negligible interference from other major species (H_2_O and CO_2_) in combustion environments. Infrared laser spectroscopy has been well developed for NH_3_ measurements at high temperature, such as 800 K,^31,32^ through careful selection of the absorption lines of NH_3_. However, in the combustion environment at a temperature, such as 1500 K, the strong absorption lines of H_2_O cannot be ignored. Besides, UV absorption spectroscopy can be cost-effective and robust. In the present work, the measurements were accomplished just with a deuterium lamp light source and a portable spectrometer. However, to manage spatially resolved quantitative measurements, UV absorption spectroscopy must be combined with other techniques, such as laser-induced fluorescence or LIPF. Moreover, it should be noted that many species, such as SO_2_,^33^ KOH, and KCl,^34^ also have strong absorption in the UV region. This might introduce interference to NH_3_ measurements. However, with the pre-knowledge of the according UV absorption spectra, the absorption feature of different molecular species can be distinguished, as reported by Weng et al.^35^ and Li et al.^36^ Therefore, acquiring accurate UV absorption spectra becomes crucial.

In the last few years, the authors’ research group has applied UV absorption spectroscopy in the quantitative investigation of the K–Cl–S chemistry of biomass thermal conversion through the measurements of KOH, KCl, SO_2_, and OH radicals.^35−37^ To achieve accuracy measurements using UV absorption spectroscopy, the absorption cross-section data of the probed spectral range are essential. Numerous researchers have focused on the UV absorption cross-section of NH_3_. However, almost all the absorption cross-section data were obtained at room temperature^38−45^ or lower.^40^ Only a few provided data at hot environments, including the work of Mellqvist et al.,^19^ who obtained the absorption spectrum of NH_3_ at 678 K, Davidson et al.,^46^ who obtained the high temperature absorption cross-sections at 193 nm using an ArF excimer laser in a shock tube at the temperature up to 3000 K, and Menon and Michel,^47^ who performed measurement at 222.5 nm, 230 nm, and 240 nm for NH_3_ heated to 2600 K in a shock tube. To have a quantitative measurement of NH_3_ in various high temperature environments, spectrally resolved accurate UV absorption cross-section data are needed.

In the present work, the investigation was conducted in a heating tube and a laminar flame burner providing NH_3_ samples at temperatures of 295–590 K and 1140–1570 K, respectively. Spectrally resolved absorption measurements between 190 nm and 230 nm were performed corresponding to the $\overset{∼}{A}{\;}^{1}A{\prime \prime }_{2}\overset{∼}{X}{\;}^{1}A{'}_{1}$ transition of NH_3_ with a progression in the ν_2_ bending mode. The spectrally resolved UV absorption cross-sections of NH_3_ at different temperatures were collected. Based on the experimental results, the rotational constants of NH_3_ were determined, which were used for the calculation of the absorption cross-sections of NH_3_ at different high temperatures using the PGOPHER software.^48^ Using UV absorption technique, the concentration of NH_3_ in the post flame zone of a premixed NH_3_–methane (CH_4_)–air flame at different equivalence ratios was determined.

## Methods

### Electrical Heating Gas Tube

**Figure 1.**
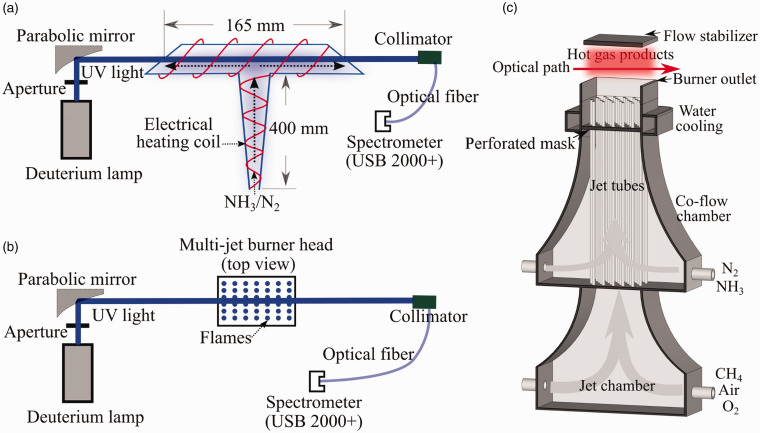
Schematic setup of broadband UV absorption spectroscopy on the (a) heating tube and the (b) multijet burner, and the (c) structure of the multijet burner.

A T-shaped electrical heated quartz tube, as shown in Fig. 1a, was used to heat a NH_3_/N_2_ gas flow to have a temperature from 295 K to 590 K. The heating tube consisted of a ∼400 mm vertical part for gas pre-heating and a horizontal part with two open ends and a length of 165 mm along the centerline for measurements, and both parts had an inner diameter of 30 mm. The total flow rate of the NH_3_/N_2_ mixture introduced into the heating tube was 20 sl/min and the NH_3_ concentration in the flow was kept at 22 ppm. Since a continuous flow with a constant speed was adopted in the present work, the effect of the adsorption/desorption of NH_3_^49–51^ on the surface of the quartz heating tube and the stainless steel gas supply tube was balanced, where the residence time of the NH_3_ flow in the heating tube is about 1 s and about 0.04 s in the gas supply tube. The measurement for each case was conducted after several minutes waiting, ensuring that the UV absorption was stabilized. An R-type thermocouple (OMEGA) with a thickness of 0.2 mm was used to measure the temperature of the gas in the center of the heating tube at different horizontal positions. The distribution of the temperature was quite even as shown in Figure S1 (Supplemental Material 1). The average temperature along the horizontal direction of the four cases adopted in the present work was 295 K, 390 K, 490 K, and 590 K, respectively.

### Burner and Flame Conditions

A multijet laminar flame burner, schematically shown in Fig. 1c, was used to provide different homogeneous hot gas environments, with varying temperature and equivalence ratio. The description of the detail of the burner has been reported by Weng et al.^52^ It consisted of two chambers, namely a jet chamber and a co-flow chamber, respectively. The premixed CH_4_–air–oxygen was introduced into the jet chamber and evenly distributed to 181 jet tubes to generate a matrix of Bunsen-type premixed flames stabilizing on each jet. The co-flow was introduced into the co-flow chamber and mixed with the hot flue gas from jet flames evenly after passing a perforated mask. After the mixing, a homogeneous hot flue gas with a certain temperature was obtained for different studies.

For the measurement of the UV absorption spectra of NH_3_ at high temperature, over 6000 ppm NH_3_ was introduced into the hot gas through the co-flow with nitrogen. The hot flue gas was provided by the flames with their conditions shown in Table I. The flames (FS1–FS4) had a constant fuel–oxygen equivalence ratio, and the generated gas products had a temperature varying from 1140 K to 1570 K at ∼5 mm above the burner outlet, which was measured by two-line atomic fluorescence (TLAF) thermometry with indium atoms with an uncertainty of ∼3%, as described in detail by Borggren et al.^53^ The fuel-rich flame cases ensured that a certain amount of NH_3_ was remained in the hot flue gas even with the consumption by active radicals, such as OH, O, and H, generated by the jet flames. Since there was more NH_3_ consumption occurring in the case with higher temperature, the introduced NH_3_ was increased from 6000 ppm to 18 000 ppm as the temperature rose from 1140 K to 1570 K. The influence of the seeding of this amount of NH_3_ on the temperature of the hot flue gas was estimated to be negligible.

**Table I.** Summary of the flame conditions adopted in this experiment with temperatures measured at 5 mm above the burner outlet.

Flame case	Gas flow rate (sl/min)						Fuel–O_2_ equivalence ratio φ	Gas product temperature (K)
	Jet flow					Co-flow		
	CH_4_	Air	O_2_	NH_3_		N_2_		
FS1	2.28	7.82	1.84	0		27.90	1.31	1140
FS2	2.47	8.47	1.99	0		22.32	1.31	1340
FS3	2.66	9.12	2.14	0		18.60	1.31	1470
FS4	3.04	10.14	2.51	0		13.95	1.31	1570
FE1	1.31	30.57	0.00	3.39		0	0.80	1750
FE2	1.47	30.57	0.00	3.80		0	0.90	1845
FE3	1.63	30.57	0.00	4.21		0	1.00	1875
FE4	1.75	30.57	0.00	4.52		0	1.07	1850
FE5	1.88	30.57	0.00	4.86		0	1.15	1825
FE6	1.99	30.57	0.00	5.14		0	1.22	1800

Moreover, premixed NH_3_–CH_4_–air flames with different equivalence ratios were also run in this burner. The UV absorption spectroscopic technique with the newly obtained absorption cross-section of NH_3_ at high temperature was adopted to measure the concentration of NH_3_ in the hot flue gas of the flames at ∼5 mm above the burner outlet. Simultaneously, based on the absorption spectra, the concentration of NO was also obtained. The flame cases (FE1–FE6) with their conditions are shown in Table I. The ratio of the volume of NH_3_ and CH_4_ was kept at ∼2.6. The equivalence ratio varied from 0.8 to 1.22, and the corresponding temperature in the center of the hot flue gas at ∼5 mm above the burner outlet is shown in Table I, which was measured by a calibrated B-type thermocouple (OMEGA) with thermal radiation loss correction based on the heat transfer theory as reported by Weng et al.^52^

### Optical System

The schematic of the broadband UV absorption spectroscopy optical system is shown in Fig. 1. A deuterium lamp (Hamamatsu Photonics) was used to generate UV light. After an aperture and a parabolic mirror, a ∼1 cm UV beam was guided through the measurement zone and collected by a spectrometer after a collimator. In the present work, two different spectrometers with different spectral resolutions, i.e., ∼0.18 nm (Andor, Model Shamrock 750, *f*/9.7, 300 lines/mm grating) and ∼1.5 nm (Ocean Optics, USB 2000+), were used. The high-resolution spectrometer had an exposure time of 10 μs and 6000 measurements were averaged, while the low-resolution spectrometer had a 3 ms exposure time and 1000 measurements were averaged.

The distribution of the NH_3_ at the edge of the heating tube at room temperature was measured using LIPF.^22^ In the present work, a 193 nm pulse laser provided by an ArF Excimer laser (Compex 102, Lambda Physik, 5 Hz, 70 mJ/pulse) was transformed into a laser sheet with a height of ∼25 mm and a thickness of around 0.5 mm. The laser sheet vertically entered the heating tube and photodissociated the NH_3_ molecules. After the photofragmentation, NH radicals in excited states were produced and the according fluorescence was collected using an intensified charge-coupled device camera with an optical bandpass filter at 336 nm with a full width half-maximum of 10 nm. The fluorescence signal is shown in Figure S2 (Supplemental Material 1). Combing the profile of the fluorescence signal and the temperature distribution shown in Figure S1, the optical path length of the measurements in the heating tube was determined to be 183 mm.

### Theory

Ultraviolet (UV) absorption spectroscopy is developed based on the Beer–Lambert law

(1) $$-\ln(I_{\text{s}}(\lambda )/I_{0}(\lambda ))=\sigma (\lambda ,T)\cdot L\cdot N$$

where –ln(*I*_s_(λ)/*I*_0_(λ)) is the absorbance, derived from the UV light intensity after the passage of the hot flue gas with and without absorbing species, i.e., *I*_s_(λ) and *I*_0_(λ), respectively; *L* is the optical path length; and *N* is the number density of the absorbing species. Thus, to quantitatively measure the concentration of NH_3_ in the environments at different temperatures, correct absorption cross-sections, σ(λ,*T*), are essential.

Moreover, to simulate the absorption spectra obtained from the experimental measurements, instrument broadening was added with a convolution of a Gaussian function, g(λ), based on the resolution of the spectrometer

(2) $$\mathrm{Ab}(\lambda )=-\text{ln}[1-(1-e^{-A(\lambda )})*g(\lambda )]$$

where *Ab*(λ) and *A*(λ) are the absorbances with and without involving the broadening effect, respectively.

Using UV absorption spectroscopy, the concentration of NO in the combustion environments was also measured, where the absorption attributed to the (0–0) vibration transition at around 226 nm was used. The absorption cross-section data was extracted from LIFBASE.^54^ For each specified transition *i* of the (0–0) vibrational band, the frequency (ν) dependent absorbance, *A_i_*(ν), was expressed as

(3) $$A_{i}(\nu )=N\cdot L\cdot B_{i}\cdot h\cdot \nu_{i}/c\cdot {\mathrm{Fb}}_{i}\cdot (\nu )$$

where *B_i_* is the absorption coefficients, *h* is the Planck constant, *c* is the speed of light, *Fb_i_* is the Boltzmann fraction, and  (ν) is the area-normalized, line-shape function. The total absorbance involving the instrument broadening effect was obtained with the equation

(4) $$\mathrm{Ab}(\nu )=-\text{ln}[\Pi (1-(1-e^{-A_{i}(\nu )})*g(\nu ))]$$

## Results and Discussion

### Absorption Cross-Section of Ammonia

**Figure 2.**
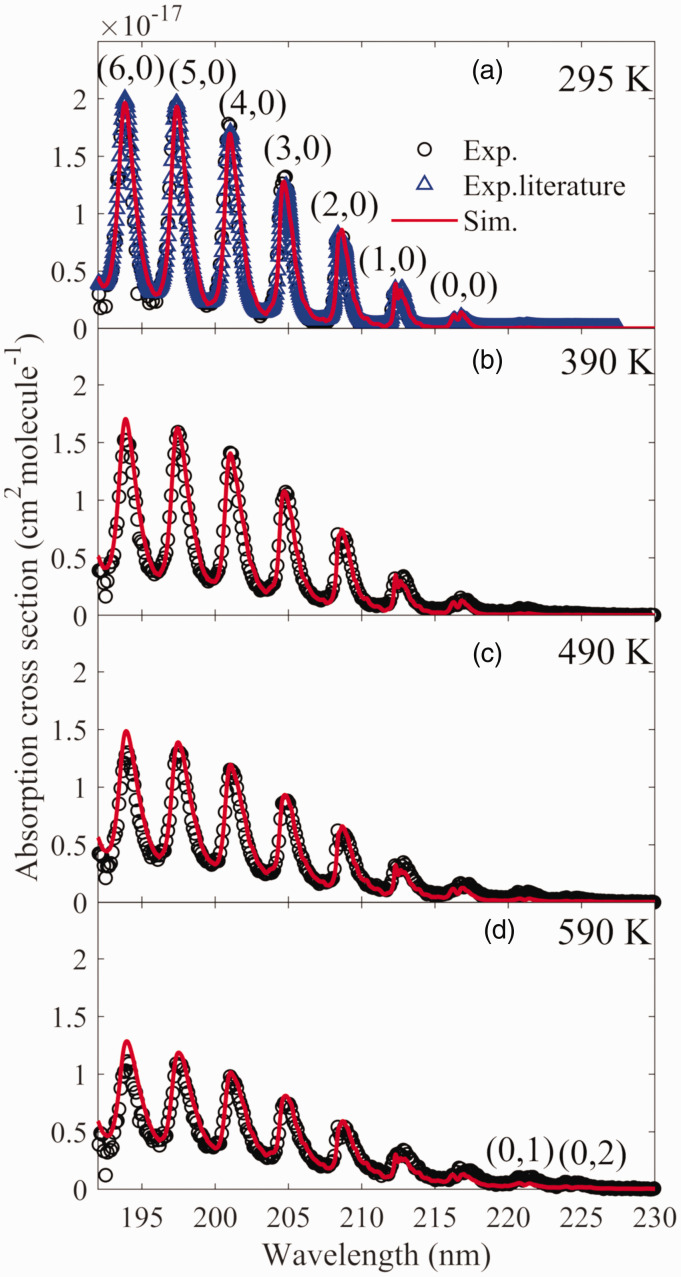
The UV absorption cross-section of ammonia as a function of wavelength obtained from the experimental measurement and the PGOPHER simulation at the temperature of (a) 295 K, (b) 390 K, (c) 490 K, and (d) 590 K.

The absorption cross-section of NH_3_ at room temperature (295 K) was derived (Fig. 2a) based on the Beer–Lambert law in which the absorbance was obtained from the experimental measurement using the high-resolution spectrometer, the NH_3_ concentration was 22 ppm, and the optical path length was 183 mm. It shows a good agreement with the most recently reported absorption cross-section data in the work of Limão-Vieira et al.^38^ The UV spectra covered the range of wavelengths from 190 nm to 230 nm. The absorption was attributed to the A∼1A″2X∼1A1' transition of NH_3_, corresponding to a progression in the umbrella bending mode, ν_2_. The absorption spectrum appeared in the form of discrete vibronic bands, spaced by approximately 900 cm^−1^, above an apparent continuum, and the details of the key features have been widely studied.^38,55^

The gas in the heating tube was heated up to 390 K, 490 K, and 590 K with a constant NH_3_ concentration. Thus, the absorption cross-section at different temperatures was derived based on the Beer–Lambert law using the measured absorbance at the corresponding temperature. As shown in Fig. 2, the absorption cross-section of NH_3_ decreased with increasing temperature, significantly for the discrete vibronic peaks, caused by the reducing population of the ground vibrational state at higher temperature. For example, the peak absorption cross-section of the (6, 0) vibrational band of NH_3_ was reduced from ∼2 × 10^−17^ to ∼1.2 × 10^−17^ cm^2^/molecule as the temperature was increased from 295 K to 590 K. Under the conditions at high temperature, the hot band absorption was observed.

The simulation of the absorption spectra of NH_3_ ($\overset{∼}{A}{\;}^{1}A{\prime \prime }_{2}\overset{∼}{X}{\;}^{1}A{'}_{1}$ transition) was performed using the PGOPHER software^48^ at different temperatures, and the rotational constants of NH_3_ were determined by fitting the simulated spectra to the experimental ones. The calculation of the absorption cross-section of NH_3_ at room temperature was first conducted. The basic constants, such as (Table II) the position of vibronic bands, the value of rotational constants in corresponding vibration level of $\overset{∼}{A}$ state, $B{'}_{v}$ and $C{'}_{v}$, the rovibronic band width and the relative transition intensity, was determined when the simulated absorption cross-sections had a good fit to the experimental ones as shown in Fig. 2a. In the present work, the value of $B{'}_{v}$ and $C{'}_{v}$, and also the constants in the lowest vibrational level of the ground electronic state *B*_0_ (9.94 cm^−1^) and *C*_0_ (6.19 cm^−1^), provided by Ziegler^55^ was used. Meanwhile, in the present work the higher order terms of the rotational energy, $D{'}_{J}$ and $D{'}_{\mathrm{JK}}$, of NH_3_ (Table II) were adjusted to ensure that the simulated results have a good fit to the experimental ones at different temperatures up to 590 K, as shown in Figs. 2b to 2d. Thus, based on the constants presented in Table II, the absorption cross-sections of NH_3_ at different high temperatures over 590 K can be obtained through the PGOPHER calculation.

**Table II.** Rotational constants and rovibronic band widths in corresponding vibration level of the $\overset{∼}{A}$ state used in the PGOPHER software.

${\nu '}_{2}$	Band origin (cm^−1^)	$B{'}_{v}$ (cm^−1^)	$C{'}_{v}$ (cm^−1^)	$D{'}_{J}$ (cm^−1^)	$D{'}_{\mathrm{JK}}$ (cm^−1^)	Band width (cm^−1^)
0	46150	9.6	4.8	0	0	45
1	47050	9.05	5.2	0.01	0.02	45
2	47950	8.5	5.4	0.02	0.025	70
3	48870	8.4	5.8	0.03	0.05	95.5
4	49785	7.8	5.6	0.04	0.05	135
5	50706	7.5	5.4	0.03	0.04	185
6	51630	6.0	8.0	0.02	0.02	245

**Figure 3.**
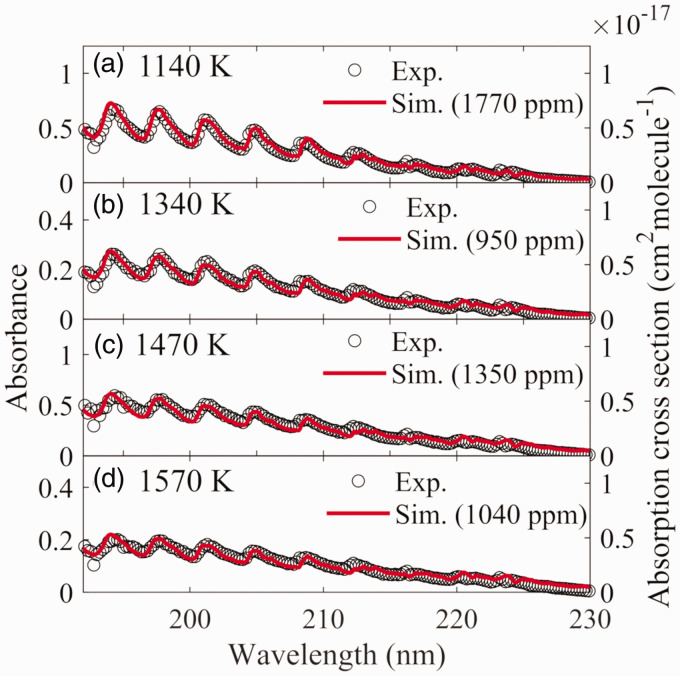
The absorbance of ammonia obtained from the experimental measurements and the simulation based on the UV absorption cross-section calculation using PGOPHER at the temperature of (a) 1140 K, (b) 1340 K, (c) 1470 K, and (d) 1570 K.

Using the multijet burner, the experimental investigation of the UV absorption spectra of NH_3_ was extended to the temperature between 1140 K and 1570 K. In the multijet burner, a certain amount of NH_3_, i.e., 6000 ppm, 7000 ppm, 12 000 ppm, and 18 000 ppm of the total flow, was introduced through the co-flow to the hot flue gas environments at 1140 K, 1340 K, 1470 K, and 1570 K, respectively. As shown in Table I, fuel rich conditions were used to provide the hot flue gas with negligible oxygen. However, it was found that the radicals, such as OH, produced by the hot premixed CH_4_ flames and present in the hot flue gas, could react with NH_3_. The NH_3_ concentration in the hot flue gas became unknown. Thus, from the measurement, only the absorbance of NH_3_ was obtained, which is presented in Fig. 3 against the left *y*-axis.

The UV absorption cross-sections at temperatures between 1140 K and 1570 K were calculated using the PGOPHER software using the constants in Table II. The results were plotted against the right *y*-axis in Fig. 3. Based on the calculated absorption cross-sections and the Beer–Lambert law, the absorbance was simulated as the concentration was set to be 1770 ppm, 950 ppm, 1350 ppm and 1040 ppm at the temperature of 1140 K, 1340 K, 1470 K, and 1570 K, respectively, and the optical path length was set to be 0.85 m.^37^ The absorbance from the simulation was plotted against the left *y*-axis in Fig. 3. As shown in Fig. 3, the profile of the spectra obtained from the simulation presented a good agreement with the results from the measurement, which indicates that the absorption cross-sections calculated by the PGOPHER software can be reliably used in the measurements at different high temperatures.

**Figure 4.**
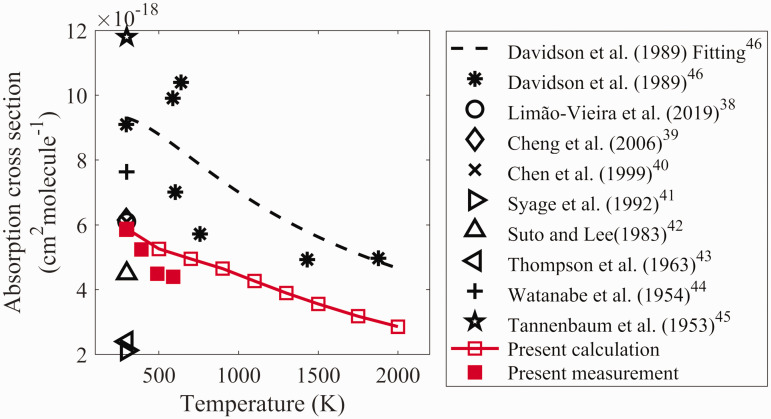
The absorption cross-section of ammonia at 193 nm as a function of temperature.

In Fig. 4, the absorption cross-sections at different temperatures obtained in the present work were compared with the ones reported in previous investigations. The temperature-dependent and spectrally resolved absorption cross-sections have not been found in literature. The only study was conducted by Davidson et al.^46^ at 193 nm from room temperature up to 3000 K, and Menon and Michel^47^ at 222.5 nm, 230 nm, and 240 nm from room temperature up to 2600 K. Here, the cross-section of NH_3_ at 193 nm was compared considering that, in the present work, the results at this wavelength were more reliable than the ones at 222.5 nm, 230 nm, and 240 nm due to a larger cross-section, and the cross-section at 193 nm is very important as 193 nm excimer laser has been widely used for NH_3_ detection using a photofragmentation process and an accurate absorption cross-section helps the quantification of these measurements. As shown in Fig. 4, both simulated and experimental measured absorption cross-sections at 193 nm obtained from the present work gradually decreased with increasing temperature, being similar to the ones presented by Davidson et al.,^46^ but with different values. The decrease was caused by the distribution of the ground state and has been fitted by Davidson et al.^46^ Due to the low sensitivity of the spectrometer at 193 nm, the high measurement uncertainty led to the divergence of the simulation and experimental results. The absorption cross-section obtained from the present work at room temperature is close to the ones from the most recent work provided by Limão-Vieira et al.^38^ and Cheng et al.,^39^ and it is believed that the values of the present work at different high temperatures also have a good confidence.

### Measurement Using Low-Resolution Spectrometer

**Figure 5.**
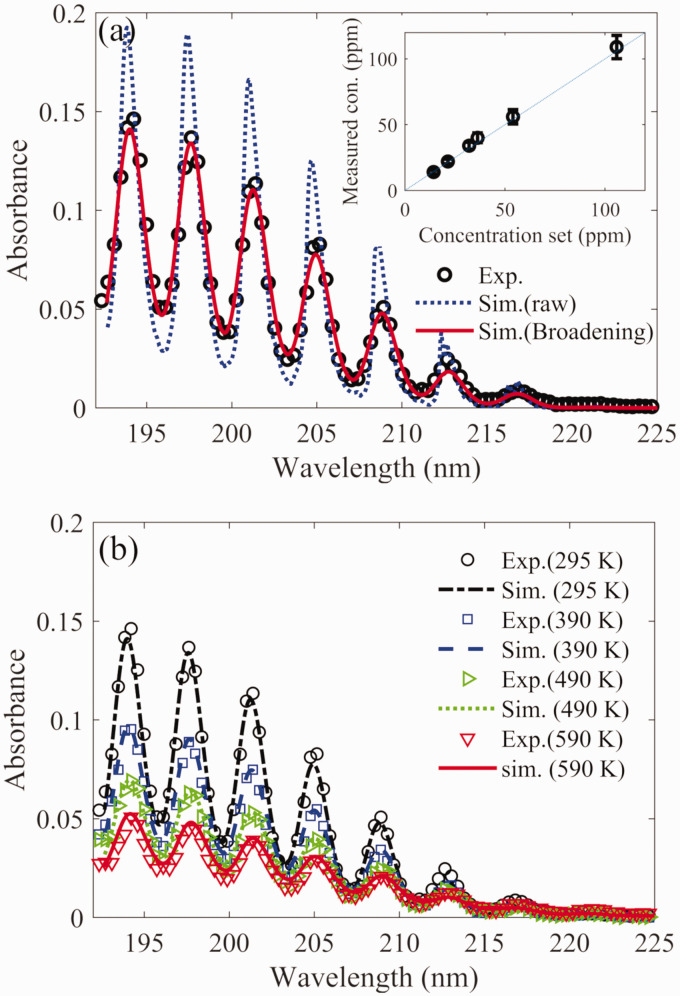
The UV absorbance as a function of wavelength obtained by the low-resolution spectrometer as 22 ppm ammonia was introduced in the heating tube at (a) room temperature and (b) different high temperatures, and corresponding calculation using the PGOPHER software with and without involving instrument broadening effect. The inset shows the measured concentration derived from the calculated absorption cross-section under the conditions with different amount of ammonia seeding at room temperature.

A portable (about 9 × 6 × 3 cm^3^) and inexpensive spectrometer (Ocean Optics, 2000+) was also adopted to achieve the NH_3_ measurement, even though it had a much lower resolution than the high-resolution spectrometer (Andor, Model Shamrock 750, *f*/9.7, 300 lines/mm grating). A typical absorption spectrum of 22 ppm NH_3_ in a nitrogen flow at room temperature measured by the low-resolution spectrometer is presented in Fig. 5a. Using the absorption cross-section of NH_3_ at room temperature, corresponding absorbance spectrum was calculated, and in the simulation of the experimental results, instrument broadening was added with a convolution of a Gaussian function having a root mean square width of 150 cm^−1^, based on Eq. 2.

The simulated spectrum involving instrument broadening is shown in Fig. 5a with a good fit to the experimental results. Through this calculation process, the concentration of NH_3_ in different gas environments can be derived. As shown in the inset of Fig. 5a, the concentration of NH_3_ can be well measured at different level of concentration with a detection limit estimated to be less than 10 ppm with an optical path length of 20 cm. Meanwhile, involving the same instrument broadening, the simulation of the absorption spectra of 22 ppm NH_3_ in different hot gas environments at a temperature up to 590 K was conducted, and shows a good agreement with the experimental results (Fig. 5b).

### Concentration Measurements of NH_3_ and NO in Flames

**Figure 6.**
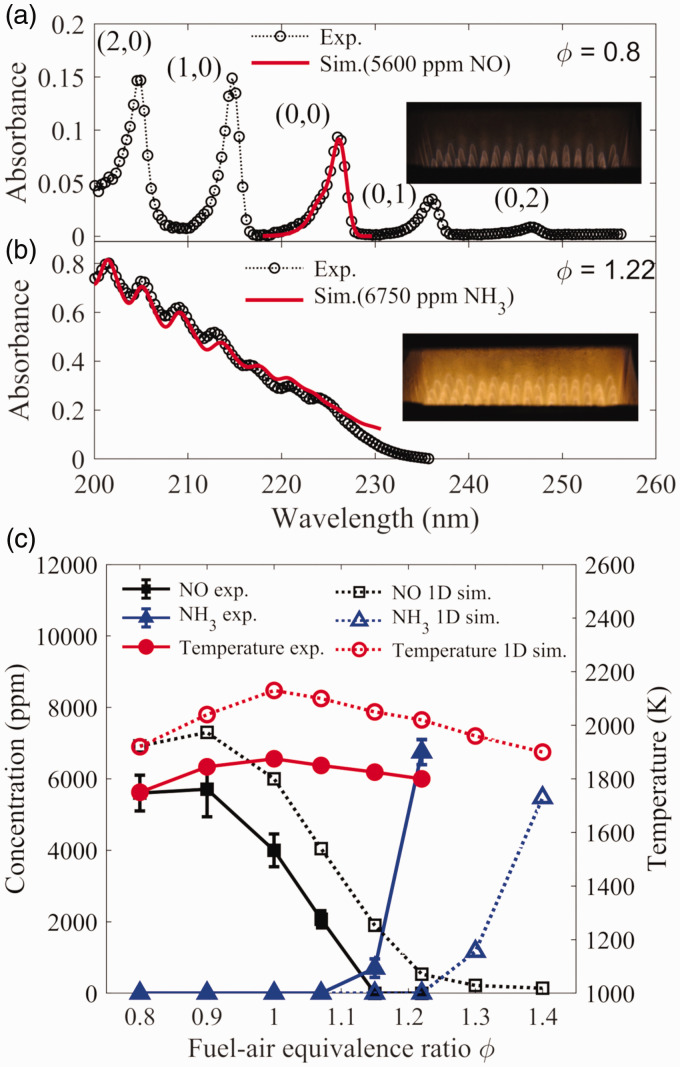
The absorption spectra of the hot gas provided by the ammonia–methane–air premixed flame at a fuel–air equivalence ratio of (a) 0.8 and (b) 1.22 with the fitting by the absorption of NO and NH_3_, and (c) the concentration of NO and NH_3_ and temperature as a function of fuel–air equivalence ratio of the flame from experimental measurements and one-dimensional simulation.

Recently, the characterization of NH_3_ combustion has become attractive as NH_3_ is regarded as a promising carbon-free energy carrier. To understand the thermochemistry of NH_3_, the measurement of nitrogen species is essential. The broadband UV absorption spectroscopy was performed to measure the concentration of NH_3_ and NO in the post flame zone of the premixed NH_3_–CH_4_–air flames provided by the multijet burner. The flame conditions were presented in Table I (FE1–FE6) with varying the fuel–air equivalence ratio from lean to rich. Lifted Bunsen-type premixed flames were generated in the burner as shown in Fig. 6. The measurement was conducted at ∼5 mm above the burner outlet with an optical path length of ∼85 mm,^37^ which was at ∼30 mm downstream of the flame fronts. The temperature at the center of the measurement region was measured by a B-type thermocouple, and it is shown in Table I. The highest temperature was obtained under the stoichiometric condition. Typical absorbance at a fuel-lean and a fuel-rich condition is presented in Figs. 6a and 6b, respectively. In the fuel-lean case, the absorption spectrum was attributed to NO present in the hot flue gas. Corresponding vibration bands from the $A{\;}^{2}\Sigma^{+}X{\;}^{2}\Pi$ transition are indicated in the figure. As shown in Fig. 6a, the simulation with an NO concentration of 5600 ppm could well fit the measured one. In the present work, using the low-resolution spectrometer, the detection limit of NO was estimated to be ∼200 ppm. When the flame was switched to the fuel-rich condition, the concentration of NO was lower than the detection limit. It should be noted that, even in the environment containing NH_3_, the measurement of NO still had a similar detection limit, since at high temperature, at the wavelength longer than 230 nm, NH_3_ only had a weak flat absorption, which made the absorption of NO be distinguishable based on the absorption of the (0, 1) vibrational band. Under the rich condition, the measured absorbance (Fig. 6b) was confirmed to be contributed by the absorption of NH_3_. Using the absorption cross-section of NH_3_ at this temperature calculated by the PGOPHER software with the constants obtained from the present work (Table II), the concentration of NH_3_ in the post flame zone was calculated to be around 6750 ppm. As presented in Fig. 6b, the absorption at the wavelength longer than 225 nm was not well fitted, which indicated that, at these wavelengths, the absorption cross-sections of NH_3_ were not well calculated at a high temperature using the rotational constants obtained from the present work. Therefore, more experimental work was demanded to determine the constants further accurately.

The variation of the concentration of NH_3_ and NO in the post flame zone as a function of the fuel–air equivalence ratio is presented in Fig. 6c together with the gas temperature. The uncertainty of the measured NH_3_ and NO concentration was originated from the curve-fitting process. As the equivalence ratio increased from 0.8 to 1.07, only NO was detected with a reduction from 5600 ppm to 2070 ppm. As the equivalence ratio increased to 1.15 and 1.22, NO concentration was barely detected, which was lower than the detection limit (∼200 ppm), but 700 ppm and 6750 ppm unburned NH_3_ were measured, respectively. The observed results were similar to the results from the previous research performed in a micro gas turbine combustor.^11^

The simulation of NH_3_–CH_4_–air premixed combustion was performed and compared with the experimental results. In the simulation, a one-dimensional free propagation premixed flame model from CHEMKIN-PRO^56^ was used, and the detailed reaction mechanism developed by Okafor et al.^57^ was adopted. The simulation results, presented in Fig. 6c, are the concentration of NO, NH_3_, and temperature at 3 cm away from the flame front which was located by the NH radical concentration peak. Since the simulation was performed under an adiabatic condition, the temperature obtained from the simulation was about 200 K higher than the experimental one. A similar variation trend of both NO and NH_3_ concentration was observed in the measurement and simulation. However, the minimum NO/NH_3_ emission point of the simulation is located near equivalence ratio around 1.25, while the measurement had the point near the equivalence ratio of 1.05. Similar difference was also observed by Hayakawa et al.,^58^ in which the experiment was conducted in a swirl combustor, and near the liner wall, some NH_3_ was unburned. In the present work, since lifted premixed flames were adopted, the NH_3_ slip might happen due to the open bottom of the lifted Bunsen-type flames and led to the difference between the simulation and experimental results.

## Conclusion

The spectrally resolved UV absorption cross-section of NH_3_ in hot gas environments was investigated, for the first time, through both experimental measurements and PGOPHER simulation. The present investigation was focused on the absorption at the wavelength from 190 to 230 nm ($\overset{∼}{A}{\;}^{1}A{\prime \prime }_{2}\overset{∼}{X}{\;}^{1}A{'}_{1}$ transition), and the temperature up to 1570 K using a heating gas tube and a multijet burner. The absorption cross-section of NH_3_ at the temperature from 295 K to 590 K was obtained directly through experimental measurements and used to determine the rotational constants of the $\overset{∼}{A}{\;}^{1}A{\prime \prime }_{2}\overset{∼}{X}{\;}^{1}A{'}_{1}$ transition of NH_3_ for absorption spectrum calculation using the PGOPHER software at different temperatures. Using the obtained absorption cross-section, the broadband UV absorption spectroscopic technique could be used for the quantitative in situ measurements of NH_3_ in hot environments. Based on the instruments used in the present work, the measurement dynamic range was estimated to be around 10–500 ppm at room temperature, and it changed with temperature to around 200–10 000 ppm at 1570 K, with an optical path length of ∼20 cm and a time resolution of few seconds. The concentration of NH_3_ and NO in the post flame zone of an NH_3_–CH_4_–air premixed flame was detected, and a clear variation of the species concentration as a function of equivalence ratio (0.8–1.22) was observed and compared with the results from a one-dimensional simulation. It shows that the quantitative measurements of NH_3_ based on UV absorption spectroscopy can be used to evaluate and support the development of the detailed chemical mechanisms of NH_3_ reactions.

## Supplemental Material

sj-pdf-1-asp-10.1177_0003702821990445 - Supplemental material for Ultraviolet Absorption Cross-Sections of Ammonia at Elevated Temperatures for Nonintrusive Quantitative Detection in Combustion Environments Click here for additional data file. Supplemental material, sj-pdf-1-asp-10.1177_0003702821990445 for Ultraviolet Absorption Cross-Sections of Ammonia at Elevated Temperatures for Nonintrusive Quantitative Detection in Combustion Environments by Wubin Weng, Shen Li, Marcus Aldén and Zhongshan Li in Applied Spectroscopy

sj-xlsx-2-asp-10.1177_0003702821990445 - Supplemental material for Ultraviolet Absorption Cross-Sections of Ammonia at Elevated Temperatures for Nonintrusive Quantitative Detection in Combustion Environments Click here for additional data file. Supplemental material, sj-xlsx-2-asp-10.1177_0003702821990445 for Ultraviolet Absorption Cross-Sections of Ammonia at Elevated Temperatures for Nonintrusive Quantitative Detection in Combustion Environments by Wubin Weng, Shen Li, Marcus Aldén and Zhongshan Li in Applied Spectroscopy

## Supplemental Material

The supplemental material mentioned in the text is available in the online version of the journal.
