# Supplementary material for: Ultraviolet Absorption Cross-Sections of Ammonia at Elevated Temperatures for Nonintrusive Quantitative Detection in Combustion Environments
Source: Appl Spectrosc. 2021 Feb 2;75(9):1168–77. doi: 10.1177/0003702821990445 (PMC8446901; doi:10.1177/0003702821990445)
Supplement: sj-pdf-1-asp-10.1177_0003702821990445 - Supplemental material for Ultraviolet Absorption Cross-Sections of Ammonia at Elevated Temperatures for Nonintrusive Quantitative Detection in Combustion Environments [file sj-pdf-1-asp-10.1177_0003702821990445.pdf]

Ultraviolet Absorption Cross-Sections of Ammonia at Elevated Temperatures for Nonintrusive Quantitative Detection in Combustion Environments

Wubin Weng*, Shen Li, Marcus Aldén and Zhongshan Li
Division of Combustion Physics, Lund University, P.O. Box 118, SE-221 00, Lund, Sweden
* Corresponding author email: wubin.weng@forbrf.lth.s3

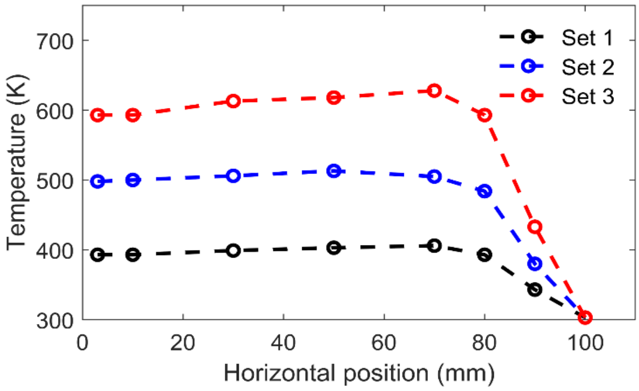

Figure S1. Distribution of the temperature along the horizontal direction of the heating tube from the center to the edge.

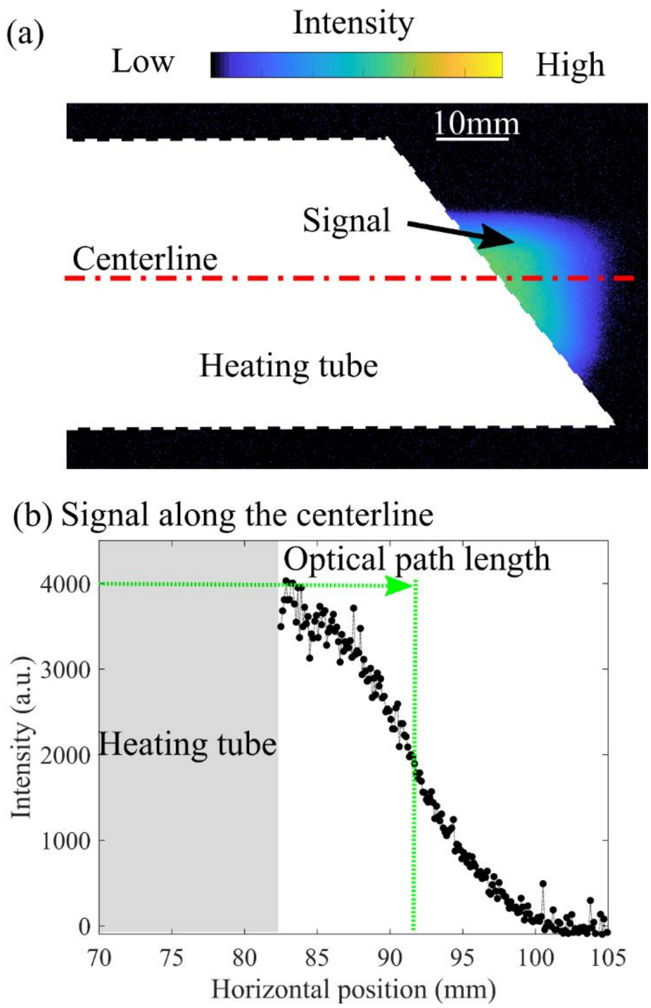

Figure S2. Distribution of the laser-induced photofragmentation fluorescence of NH_3 (a) and the profile of the signal along the centerline of the heating tube.
